# Digital outcome measures are associated with brain atrophy in patients with multiple sclerosis

**DOI:** 10.1007/s00415-024-12516-9

**Published:** 2024-07-15

**Authors:** Pam C. G. Molenaar, Samantha Noteboom, David R. van Nederpelt, Eva A. Krijnen, Julia R. Jelgerhuis, Ka-Hoo Lam, Gerrieke B. Druijff-van de Woestijne, Kim A. Meijer, Pim van Oirschot, Brigit A. de Jong, Iman Brouwer, Bas Jasperse, Vincent de Groot, Bernard M. J. Uitdehaag, Menno M. Schoonheim, Eva M. M. Strijbis, Joep Killestein

**Affiliations:** 1 MS Center Amsterdam, Neurology, Vrije Universiteit Amsterdam, Amsterdam Neuroscience, Amsterdam UMC Location VUmc Polikliniek Neurologie, Attn. MS Center Amsterdam, P. O. Box 7057, 1007 MB Amsterdam, The Netherlands; 2 MS Center Amsterdam, Anatomy and Neurosciences, Vrije Universiteit Amsterdam, Amsterdam Neuroscience, Amsterdam UMC Location VUmc, Amsterdam, The Netherlands; 3 MS Center Amsterdam, Radiology and Nuclear Medicine, Vrije Universiteit Amsterdam, Amsterdam Neuroscience, Amsterdam UMC Location VUmc, Amsterdam, The Netherlands; 4 Neurocast B.V., Amsterdam, The Netherlands; 5 Sherpa B.V., Amsterdam, The Netherlands; 6 MS Center Amsterdam, Rehabilitation Medicine, Vrije Universiteit Amsterdam, Amsterdam UMC Location VUmc, Amsterdam, The Netherlands

**Keywords:** Multiple sclerosis, Outpatient monitoring, Smartphone, Atrophy, Cognition

## Abstract

**Background:**

Digital monitoring of people with multiple sclerosis (PwMS) using smartphone-based monitoring tools is a promising method to assess disease activity and progression.

**Objective:**

To study cross-sectional and longitudinal associations between active and passive digital monitoring parameters and MRI volume measures in PwMS.

**Methods:**

In this prospective study, 92 PwMS were included. Clinical tests [Expanded Disability Status Scale (EDSS), Timed 25 Foot Walk test (T25FW), 9-Hole Peg Test (NHPT), and Symbol Digit Modalities Test (SDMT)] and structural MRI scans were performed at baseline (M0) and 12-month follow-up (M12). Active monitoring included the smartphone-based Symbol Digit Modalities Test (sSDMT) and 2 Minute Walk Test (s2MWT), while passive monitoring was based on smartphone keystroke dynamics (KD). Linear regression analyses were used to determine cross-sectional and longitudinal relations between digital and clinical outcomes and brain volumes, with age, disease duration and sex as covariates.

**Results:**

In PwMS, both sSDMT and SDMT were associated with thalamic volumes and lesion volumes. KD were related to brain, ventricular, thalamic and lesion volumes. No relations were found between s2MWT and MRI volumes. NHPT scores were associated with lesion volumes only, while EDSS and T25FW were not related to MRI. No longitudinal associations were found for any of the outcome measures between M0 and M12.

**Conclusion:**

Our results show clear cross-sectional correlations between digital biomarkers and brain volumes in PwMS, which were not all present for conventional clinical outcomes, supporting the potential added value of digital monitoring tools.

**Supplementary Information:**

The online version contains supplementary material available at 10.1007/s00415-024-12516-9.

## Introduction

Multiple sclerosis (MS) is a neuroinflammatory and neurodegenerative disease that necessitates continuous monitoring to make adequate treatment decisions [1]. In the present-day clinical practice, the main focus of MS care is on monitoring relapses and active inflammation through MRI scans and to subsequently prevent short and long-term disability by suppressing this inflammation [2, 3]. Nevertheless, in addition to focal inflammatory processes, neurodegeneration is thought to be the main driver of slow disease progression, leading to irreversible disability with high impact on day-to-day functioning of people with MS (PwMS) [4]. As such, brain atrophy measurements on structural MRI scans are an increasingly important outcome to quantify the severity of neurodegeneration, but accurate assessment on the patient level is currently not feasible using clinical routine scans due to high variability between scanners and repeated measures [5, 6]. In clinical practice and trials, the most widely used clinical outcomes include the Expanded Disability Status Scale (EDSS), Timed 25 Foot Walk test (T25FW), 9-Hole Peg Test (NHPT), and Symbol Digit Modalities Test (SDMT). [7, 8] However, these clinical assessments are constrained by their reliance on periodic clinical appointments and are also criticized for their noise and limited sensitivity in capturing disease progression. [8, 9]

Digital smartphone-based tests offer a promising approach to monitor the presence and progression of MS symptoms. Digital outcomes have the advantage that they can be measured with higher frequency and in the patients’ own environment. As such they might capture other, perhaps more subtle aspects of MS related progression compared to conventional outcome measures. Monitoring with these tests has been applied in MS both through active and passive data collection [10–13]. Examples of active tests include the smartphone-based 2 Minute Walk Test (s2MWT) as a measure of walking ability, Draw a Shape Test and Pinching Test to assess upper extremity function and the smartphone-based SDMT (sSDMT) as a measure of cognition [14–16]. Possible passive monitoring approaches are collection of gait and mobility behavior or capturing keystroke dynamics (KD) during daily smartphone life [17, 18]. Previous research has shown that s2MWT, sSDMT and keystroke dynamics were associated with conventional clinical outcomes and cognition [19–22].

Until now, only a few studies have demonstrated cross-sectional relations between digital measures and brain atrophy [14, 23, 24]. However, the relation between KD and brain atrophy remains unexplored, and to our knowledge, no other studies have investigated the longitudinal associations between digital outcomes and MRI brain volumes so far.

Here, we studied cross-sectional and longitudinal associations between previously validated actively and passively collected smartphone-based digital outcome measures (sSDMT, 2MWT and KD [16, 19, 20]) and MRI-derived brain volume measures (brain, ventricles, cortex, thalamus and lesions) in PwMS over a 1-year follow-up. Additionally, we assessed associations between these brain volume measures and conventional clinical outcome measures (EDSS, T25FW, SDMT and NHPT). Finally, we studied if baseline brain volume measures and lesion volume outcomes are predictive of 1-year changes in clinical and digital outcome measures in PwMS.

## Methods

### Population and data collection

PwMS and healthy controls (HC) were included from the ‘Assessing fatigue, disease Activity and Progression through smartphone Surveillance in Multiple Sclerosis’ (APPS-MS) study, a 1-year prospective cohort study at the MS Center Amsterdam, Amsterdam UMC, location VUmc from August 2018 to January 2021. Written informed consent was collected prior to inclusion.

The study design and prior analyses regarding associations between clinical and digital outcomes in this cohort were reported previously [17, 19, 20]. Briefly, participants were between 18 and 65 years, reported regular use of a smartphone, and had no impairments in vision or upper limb function that interfered with typical smartphone usage. Additional eligibility criteria for PwMS included a diagnosis of relapsing–remitting multiple sclerosis (RRMS), secondary progressive multiple sclerosis (SPMS), or primary progressive multiple sclerosis (PPMS) according to the revised McDonald 2017 criteria [25]. Exclusion criteria were an EDSS ≥ 7.5 at baseline, start or switch of disease-modifying therapy (DMT) 2 months prior to inclusion, clinically relevant visual disturbances and current relevant mood-, behavioral- or sleeping disorders. PwMS with concomitant neurological or systemic disorders affecting the central nervous system were excluded from this study. In addition, participants were only included in the present study if an MRI exam containing 3D-T1 and 3D-Fluid-Attenuated Inversion Recovery (3D-FLAIR) were available at baseline (M0).

EDSS, NHPT (average dominant and non-dominant hand), T25FW and (oral) SDMT, as well as 3D-T1 and 3D-FLAIR MRI scans were collected at M0 and after 1-year follow-up (M12) for PwMS. HC underwent scanning exclusively at M0, during which their 3D-T1 and 3D-FLAIR scans were obtained.

### Digital outcomes

**Fig. 1:**
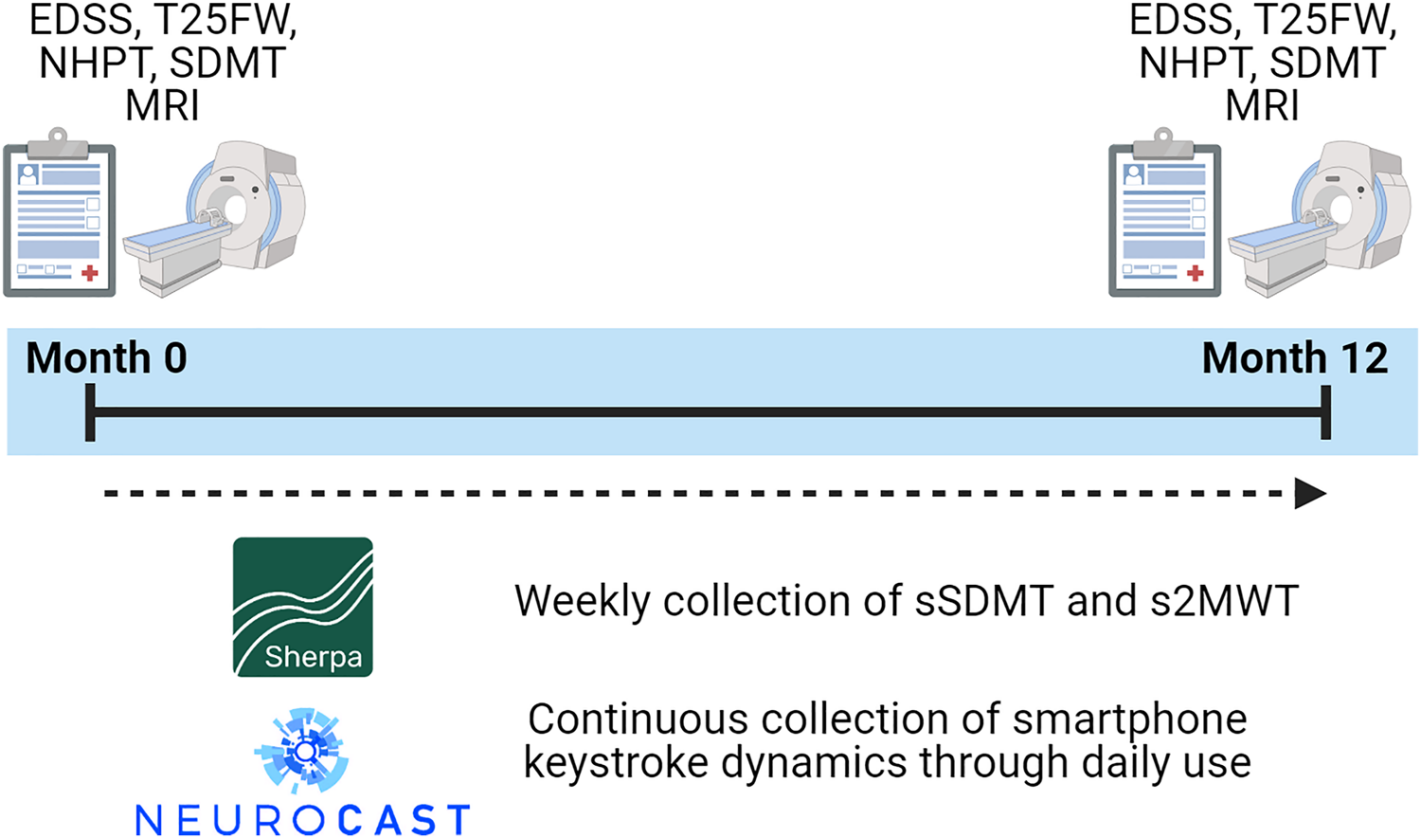
Study scheme and data collection. Clinical tests were performed at baseline (Month 0) and follow-up (Month 12). sSDMT and s2MWT were collected weekly and keystroke dynamics were collected continuously through daily smartphone use. Image created with BioRender.com. *sSDMT* smartphone-based Symbol Digit Modalities Test, *s2MWT* smartphone-based 2 Minute Walk Test, *EDSS* Expanded Disability Status scale, *T25FW* Timed 25 Foot Walk test, *NHPT* 9-Hole Peg Test, *SDMT* Symbol Digit Modalities Test

Four digital outcome measures were studied, which were collected actively or passively during 1-year follow-up [17, 19, 20]. See Fig. 1 for a schematic overview of the study schedule and data collection. The actively collected digital outcomes were gathered on a weekly basis and included the sSDMT and s2MWT. Passive monitoring of smartphone typing events was performed continuously and was used to derive a fine motor score cluster (FMSC) and cognition score cluster (CSC). Table 1 shows an overview of these outcome measures.

**Table 1:** Overview of digital outcome measures used in this study

	Construct	Data collection	Scoring
s2MWT	Walking ability	Actively	Covered walking distance in meters within 2 min. Lower scores correspond to higher levels of disability.
sSDMT	Cognition	Actively	Number of correct digit-symbol combinations within 90 s. Lower scores correspond to higher levels of disability.
FMSC	Manual dexterity	Passively	Composite score based on timing-related (press-press latency, release-release latency, flight time) keystroke features. Higher scores correspond to higher levels of disability.
CSC	Cognition	Passively	Composite score based on error-related (precorrection slowing, correction duration, postcorrection slowing) and paralinguistict (after-punctuation pause) keystroke features. Higher scores correspond to higher levels of disability.

*s2MWT* smartphone-based 2 Minute Walk Test, *sSDMT* smartphone-based Symbol Digit Modalities Test, *FMSC* fine motor score cluster, *CSC* cognition score cluster

*Active monitoring*: The MS sherpa® (Sherpa, Nijmegen, The Netherlands) app enables PwMS to self-administer the s2MWT and sSDMT, using their own smartphone device in their own environment, measuring walking ability and cognition, respectively [15, 16]. The frequency of tests can be set according to the patients or health care providers wishes, but for the APPS-MS study, they were scheduled weekly.

*Passive monitoring*: Data on KD during daily life phone use by PwMS were collected continuously through the Neurokeys® keyboard (Neurocast, Amsterdam, The Netherlands), installed on the patients’ smartphone. From these data, KD are derived and aggregated per day, from which the fine motor score cluster (FMSC) and cognition score cluster (CSC) were composited, measuring manual dexterity and cognition, respectively [17, 22].

For all digital outcome measures, the median values of scores collected during the first 14 days of the study were calculated for cross-sectional comparisons. Longitudinal changes in digital outcome measures over the 1-year follow-up were calculated by subtracting the median value from the last 14 days of our study by the median of the first 14 days of the study.

### MRI acquisition

MRI acquisition was performed at M0 and M12 on a 3 T GE Discovery MR750 (GE Healthcare, Milwaukee, USA) with an 8-channel head coil. All scans in this study were acquired using the same scanner. The scanner protocol included a high-resolution, 3D-T1 fast spoiled gradient-echo sequence for volumetric measurements (repetition time 8.2 ms, echo time 3.2 ms, inversion time 450 ms, flip angle 12°, voxel size: 0.9 × 0.9 × 1.0mm^3^) and a 3D-FLAIR sequence (repetition time 8000 ms, echo time 129 ms, inversion time 2340 ms, voxel size 1.0 × 1.0 × 1.2 mm^3^).

### MRI analysis

White matter (WM) lesions were automatically segmented on 3D-FLAIR (registered to 3D-T1 with ANTs) and 3D-T1 with NicMSLesions V0.2 and manually corrected [26]. To minimize the effect of lesions on brain segmentation, lesions were filled with the NiftySeg package on 3D-T1 based on the lesion masks [27]. Subsequently, whole-brain, ventricle, cortex and thalamic volumes at baseline were derived with Sequence Adaptive Multimodal SEGmentation SAMSEG (part of FreeSurfer v7.3.2) on the lesion-filled 3D-T1 [28]. All MRI volumes, except for lesion volumes, were normalized for head size by dividing them by the segmentation-based total intracranial volume (SBTIV). To calculate longitudinal 1-year atrophy rates, the longitudinal version of SAMSEG was used on scans acquired at M0 and M12 [29].

### Statistical analysis

All analyses were performed with R version 4.2.1 and Pingouin 0.5.2 in Python 3.9.15 [30]. Normality of variables was assessed with Kolmogorov–Smirnov tests and visual inspection of the histograms. At M0, digital outcomes (sSDMT, 2MWT, FMSC, CSC) were compared between PwMS and HC with an independent samples t test for normally distributed continuous variables and a Mann–Whitney U test for non-normally distributed continuous variables. In PwMS, M0 and M12 clinical (EDSS, T25FW, NHPT and SDMT) and digital outcomes were compared using a paired t test for normally distributed continuous variables and a Wilcoxon signed-rank test for non-normally distributed continuous variables. In addition, clinically significant deterioration in clinical outcome measures was determined between M0 and M12, using an increase in EDSS score of 1.5, 1.0 or 0.5 in the case of a baseline EDSS score of 0, 1.0–5.5 or ≥ 6.0, respectively, a cut-off of 20% negative change for the T25FW and NHPT, decline of 8 or more points for the SDMT [8, 31, 32]. To investigate if PwMS with complete digital outcome measures differed from the ones with missing data, a missing data analysis was performed for each digital monitoring outcome at M0 and M12, comparing demographical and clinical variables. A more extensive description can be found in the supplementary materials.

To determine if there was brain atrophy present in our MS cohort, MRI volumes were compared between PwMS and HC, using one-way analysis of covariance (ANCOVA) with age and sex as covariates. 1-year MRI volume changes were expressed as % change from M0 volume. A paired samples t test or Wilcoxon test was used to assess significant change between M0 and M12 MRI volumes.

To study relations between brain volume measures and digital and clinical outcome measures within PwMS, linear regression analyses were used. Non-normally distributed variables were log-transformed prior to the linear regression analyses. In all the analyses, the digital and clinical outcome measures were used as dependent variables, the brain volume measures were used as independent variable, and age, disease duration, and sex were used as covariates. Resulting regression coefficients are presented as standardized β coefficients (std. β) with standard errors. For the cross-sectional analyses, we used MRI volume and clinical outcome values collected at the M0 study visit and median digital outcome values calculated from the first 14 days of our study. To evaluate the longitudinal association between change in digital and clinical outcome measures and MRI volume changes, clinical and digital change were calculated by subtracting values collected at M12 from M0. Only the regions for which MRI volumes showed a significant change between M0 en M12 were evaluated in the longitudinal regression analysis. Finally, the predictive value of cross-sectional brain volumes to 1-year changes in clinical and digital outcomes was assessed with linear regression. Within the three performed linear regression analyses (cross-sectional, longitudinal and prediction analysis), *p* values of MRI volumes were adjusted for multiple testing using the Benjamini–Hochberg method to control the False Discovery Rate (FDR) [33]. Significance was determined at an FDR-adjusted *p* value < 0.05.

## Results

### Demographic, clinical, digital and MRI characteristics

A total of 102 PwMS and 24 HC were eligible for inclusion, of which 10 PwMS were excluded due to incomplete MRI scans at M0. As a result, the study included 92 PwMS and 24 HC. Table 2 shows the demographics, clinical outcomes and digital outcomes of PwMS at M0 and M12, as well as the demographics and digital outcomes at M0 for the HC. Of all patients who were not using DMT at M0, 24/37 were diagnosed with SPMS or PPMS. Among the PwMS group, 74/92 individuals had both clinical data and MRI scans available at the M12 visit. Of the digital outcome measures, some were missing at M0, mostly due to technical difficulties: s2MWT (18/92), sSDMT (7/92), FMSC (1/92) and CSC (2/92). At M12, more data were missing resulting from a lack of adherence: s2MWT (27/74), sSDMT (12/74), FMSC (18/74) and CSC (18/74). PwMS with missing CSC and FMSC at M12 were older (52.2 years vs. 45.6 years, *p* = 0.01) and scored worse on the M12 SDMT (56.1 vs. 63.1, *p* = 0.02). The full results of the missing data analyses can be found in supplementary tables 1–4. PwMS performed worse on the sSDMT, FMSC and CSC compared with HC at M0 (*p* < 0.001), but similar on the s2MWT. Between M0 and M12, 13/74 PwMS progressed based on EDSS, 8/72 on T25FW, 1/74 on NHPT and 0/74 on SDMT. PwMS showed an improvement in sSDMT, FMSC and CSC scores during the 1-year follow-up and no changes in s2MWT.

**Table 2:** Demographics, clinical outcomes, digital outcomes and MRI volumes of people with multiple sclerosis (PwMS) and healthy controls (HC) at baseline (M0) and at 1-year follow-up (M12) for PwMS

Variable	M0			M12	
	HC, *N* = 24	MS, *N* = 92	*p* value^a^	MS, *N* = 74	*p* value^b^
Follow-up interval in years					
Mean (SD)				1.1 (0.10)	
Age in years					
Median (IQR)	47.0 (26.8, 56.0)	48.5 (38.8, 54.3)	0.300	49.0 (39.3, 54.0)	
Sex, *N* (%)					
Male/female	11/13	25 (27%)/67 (73%)	0.088	22 (30%)/52 (70%)	
Subtype, *N* (%)					
RRMS/SPMS/PPMS		55 (60%)/27 (29%)/10 (11%)		41 (55%)/23 (31%)/10 (14%)	
Duration of disease in years					
Mean (SD)		9.2 (8.2)		9.5 (8.7)	
Use of DMT, *N* (%)					
High efficacy/low efficacy/no DMT		12 (13%)/43 (47%)/37 (40%)		7 (10%)/35 (47%)/32 (43%)	
Clinical disease activity^c^, *N*					
Relapse		5			
Steroid treatment		2			
EDSS		*N* = 92		*N* = 74	
Median (IQR)		3.5 (2.5, 4.0)		3.5 (2.5, 4.5)	0.600
Significant change, *N*				13	
T25FW in seconds		*N* = 89		*N* = 72	
Mean (SD)		5.39 (2.04)		5.67 (2.44)	0.072
Significant change, *N*				8	
NHPT in seconds		*N* = 91		*N* = 74	
Mean (SD)		22.68 (6.09)		21.91 (6.55)	< 0.001
Significant change, *N*				1	
SDMT		*N* = 85		*N* = 74	
Median (IQR)		55 (48, 62)		60 (52, 69)	< 0.001
Significant change, *N*				0	
s2MWT in meters	*N* = 19	*N* = 74		*N* = 47	
Mean (SD)	132.59 (46.81)	127.64 (43.51)	0.700	149.62 (39.32)	0.019
sSDMT	*N* = 24	*N* = 85		*N* = 62	
Mean (SD)	49.88 (7.64)	43.65 (7.77)	< 0.001	52.40 (8.00)	< 0.001
FMSC	*N* = 24	*N* = 91		*N* = 56	
Mean (SD)	0.32 (0.10)	0.44 (0.17)	< 0.001	0.40 (0.17)	0.009
CSC	*N* = 24	*N* = 90		*N* = 56	
Mean (SD)	0.65 (0.26)	0.92 (0.34)	< 0.001	0.82 (0.35)	< 0.001
MRI volumes	*N* = 24	*N* = 92		*N* = 74	
Brain, mean (SD), % Mean (SD)	0.71 (0.02)	0.69 (0.02)	0.002	–0.16 (0.48) %	0.007
Ventricle median (IQR), % Mean (SD)	0.016 (0.014–0.020)	0.019 (0.016–0.023)	0.009	2.2 (2.7) %	< 0.001
Cortex, mean (SD), % Mean (SD)	0.32 (0.02)	0.32 (0.01)	< 0.001	–0.11 (0.77) %	0.225
Thalamus, mean (SD), % Mean (SD)	0.0083 (0.0004)	0.0078 (0.0007)	0.005	–0.52 (0.66) %	< 0.001
Lesions (mL), median (IQR), % Mean (SD)	0.88 (0.0–1.34)	5.0 (2.1–11.5)	< 0.001	4.4 (34.2) %	0.255

^a^*p* value between PwMS and HC at M0. ^b^*p* value for changes within PwMS between M0 and M12. ^c^Clinical disease activity in 3 months prior to M0 visit

Normally distributed continuous variables were listed by the mean and standard deviation (SD). For non-normally distributed variables, the median and interquartile range (IQR) was provided. The cut-off values for a clinically meaningful change were set at an increase of 1.5, 1.0 or 0.5 in the case of a baseline EDSS score of 0, 1.0–5.5 or ≥ 6.0, respectively, a negative change of 20% for T25FW and NHPT and a negative change of 8 points for SDMT. MRI volumes at M0 are expressed as a fraction of intracranial volume (ICV), except for lesions (in mL). MRI volume changes are expressed as percentage change (%) between M0 and M12 with standard deviation (SD). Analysis of covariance demonstrates differences in MRI- and lesion volumes between HC and PwMS at M0, corrected for age and sex. Brain, thalamic and ventricular volumes differed between M0 and M12 in PwMS, no differences were found for cortical and lesion volume changes

*HC* Healthy Controls, *PwMS* people with multiple sclerosis, *RRMS* relapsing–remitting multiple sclerosis, *SPMS* secondary progressive multiple sclerosis, *PPMS* primary progressive multiple sclerosis, *DMT* disease-modifying therapy, *EDSS* Expanded Disability Status Scale, *T25FW* Timed 25 Foot Walk test, *NHPT* 9-Hole Peg Test, *SDMT* Symbol Digit Modalities Test, *s2MWT* smartphone-based 2 Minute Walk Test, *sSDMT* smartphone-based SDMT, *CSC* cognition score cluster, *FMSC* fine motor score cluster

Brain, cortex, thalamus and ventricle volumes were different between PwMS and HC, indicating the presence of atrophy in PwMS at M0 (Table 2). In PwMS, brain and thalamus volume decreased over the 1-year follow-up (–0.16 ± 0.48%, *p* = 0.007 and –0.52 ± 0.66%, *p* < 0.001, respectively) and ventricle volumes increased (2.2 ± 2.7%, *p* < 0.001).

### Cross-sectional relationships between clinical, digital and MRI measures

**Fig. 2:**
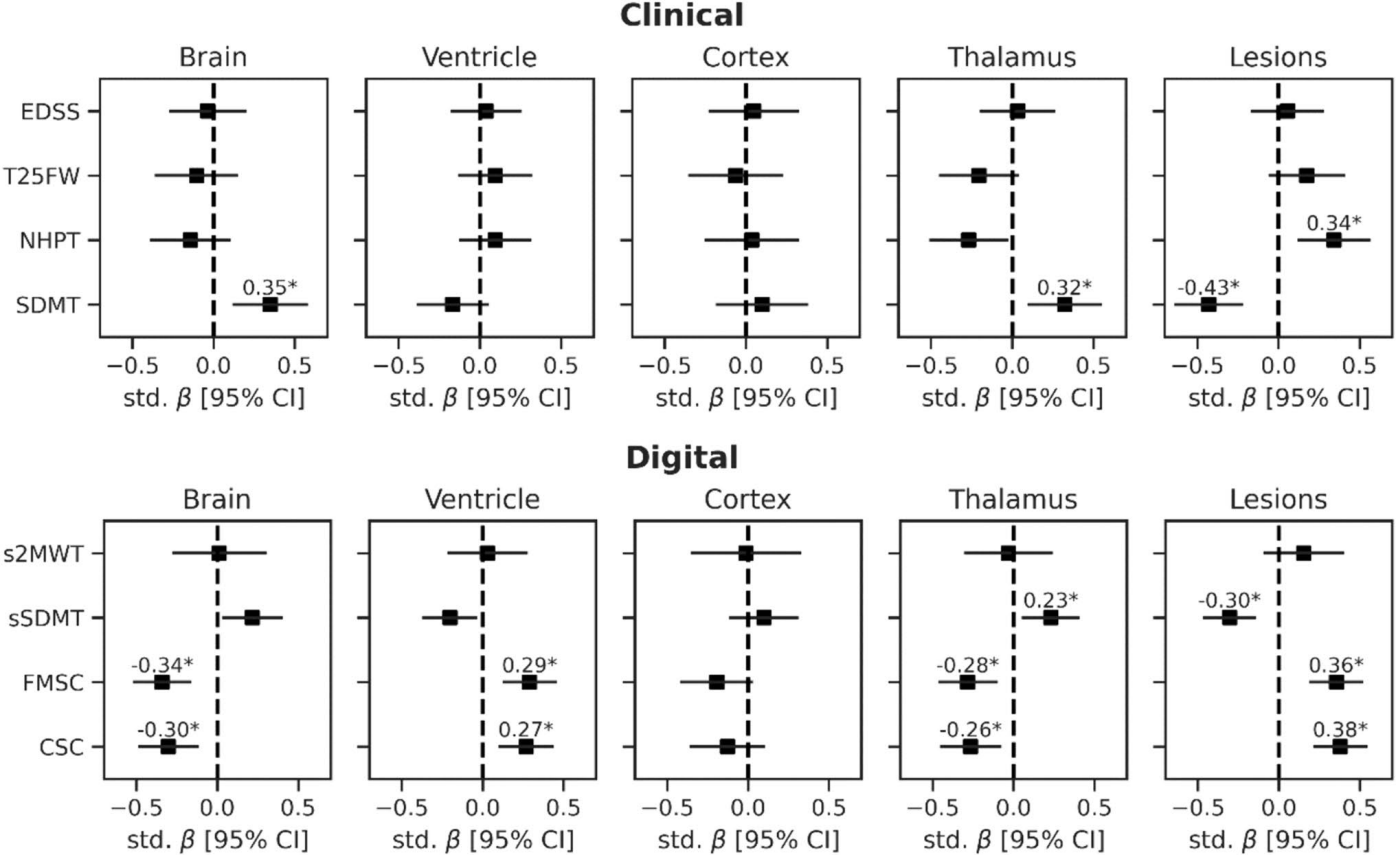
Cross-sectional relations between brain- and lesion volumes and clinical and digital outcomes in PwMS. Plots show the standardized regression coefficients (std. β) and 95% confidence intervals, corrected for age, sex and disease duration. Lesion and ventricle volumes were log-transformed. *p value < 0.05, corrected for multiple testing

Relations between clinical and digital outcomes and MRI volumes are shown in Fig. 2. The passively collected digital outcomes (FMSC, CSC) were both related to brain (std. *β* = –0.34, *p* = 0.003; std. *β* = –0.30, *p* = 0.010), ventricle (std. *β* = 0.29, *p* = 0.006; std. *β* = 0.27, *p* = 0.010), thalamus (std. *β* = –0.28, *p* = 0.012; std. *β* = –0.26, *p* = 0.020) and lesion volumes (std. *β* = 0.36, *p* = 0.001; std. *β* = 0.38, *p* < 0.001), for FMSC and CSC, respectively. From the actively collected digital outcomes, lower sSDMT scores were associated with lower thalamic volumes (std. *β* = 0.23, *p* = 0.034) and higher lesion volumes (std. *β* = –0.30, *p* = 0.003). Moreover, a trend was observed between the sSDMT and brain- (std. *β* = 0.22, uncorrected *p* = 0.022), and ventricle volume (std. *β* = –0.20, uncorrected *p* = 0.020), but did not survive correction for multiple comparisons (*p* = 0.056 and *p* = 0.053, respectively). No association between s2MWT and MRI volumes was observed. From the clinical outcomes, worse NHPT scores were associated with higher lesion volume (std. *β* = 0.34, *p* = 0.012), while worse SDMT scores were associated with higher lesion volume (std. *β* = –0.43, *p* = 0.001), as well as lower brain (std. *β* = 0.35, *p* = 0.013), and thalamic volumes (std. *β* = 0.32, *p* = 0.020). The EDSS and T25FW did not show an association with MRI volumes.

### Longitudinal relationships between clinical, digital and MRI measures

**Fig. 3:**
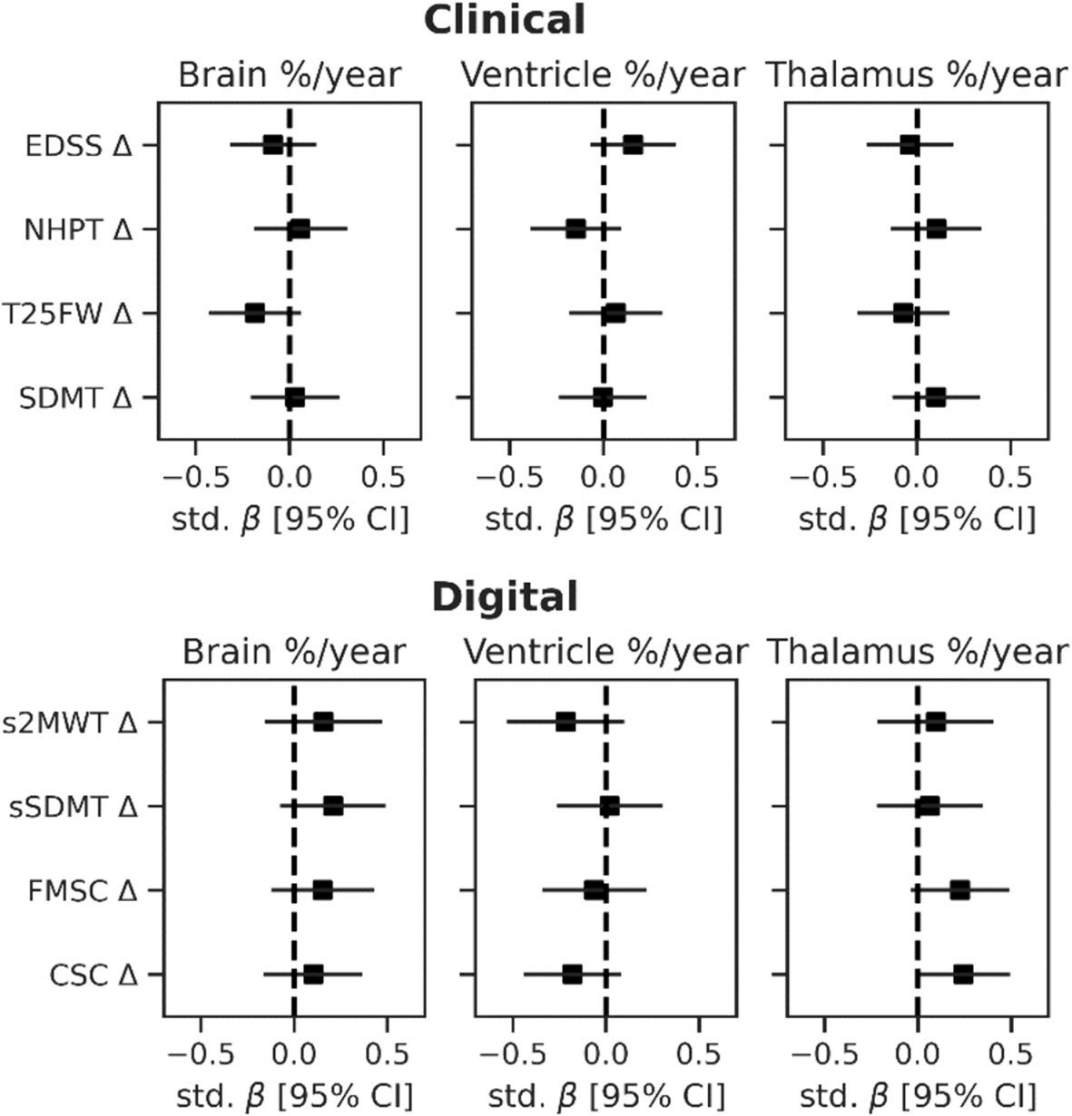
Longitudinal relations between brain and lesion volume changes and changes in clinical and digital outcomes in PwMS over 1 year. Plots show the standardized regression coefficients (std. β), corrected for age, sex and disease duration

As shown in Table 2, PwMS showed an improvement in NHPT, SDMT, sSDMT, s2MWT, FMSC and CSC scores during the 1-year follow-up and remained stable on the EDSS and T25FW. From the MRI volumes, brain and thalamus volume decreased (–0.16% and –0.52%, respectively) and ventricle volumes increased (2.2%). No significant relations were found between longitudinal changes in clinical and digital outcomes and MRI volume changes (Fig. 3). A trend was observed between changes in thalamus volume and FMSC (std. *β* = 0.23, uncorrected *p* = 0.092) and CSC (std. *β* = 0.24, uncorrected *p* = 0.057).

**Fig. 4:**
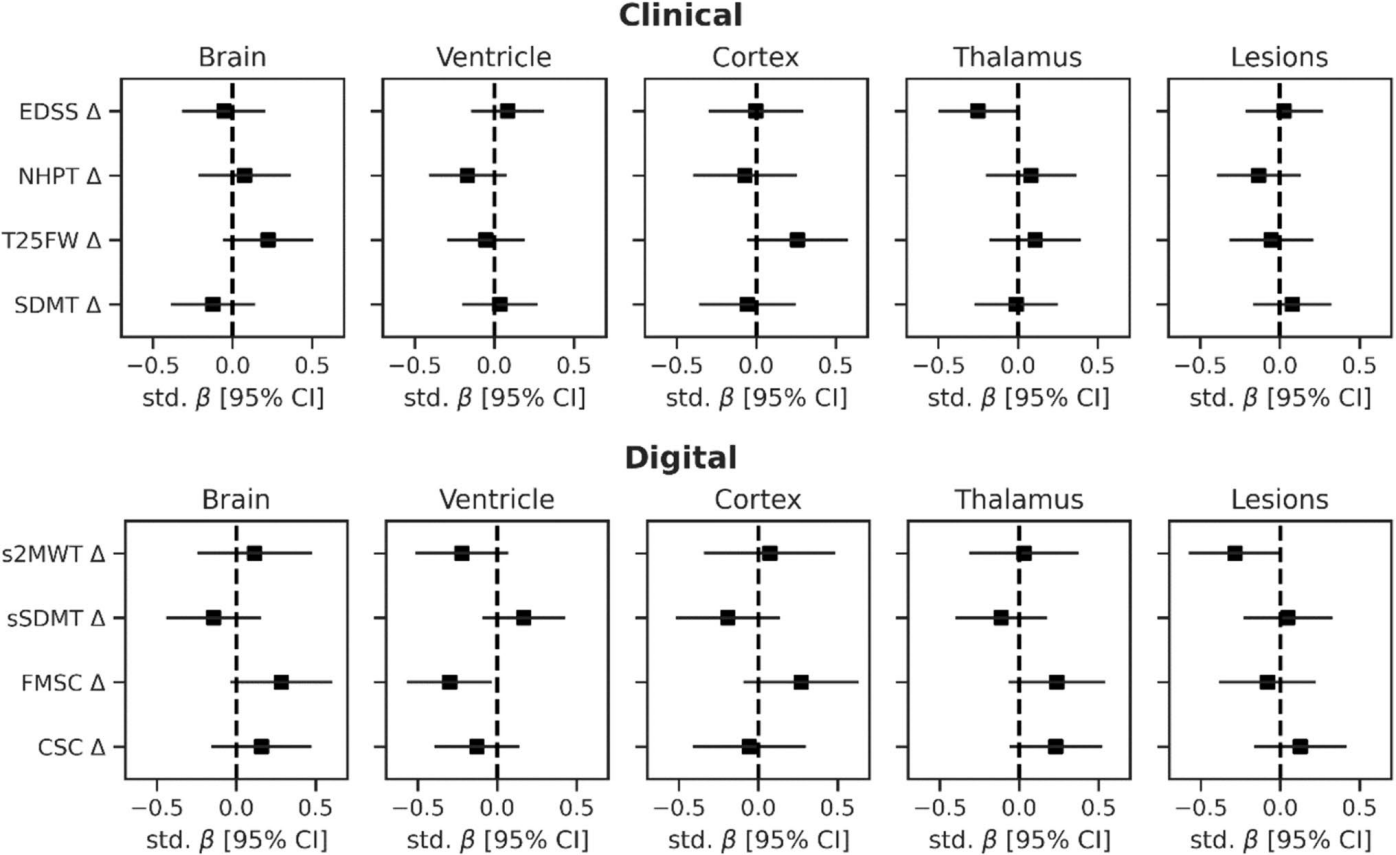
Prediction of changes in clinical and digital outcomes in PwMS over 1 year with brain and lesion volumes at baseline. Plots show the standardized regression coefficients (std. β), corrected for age, sex and disease duration

MRI volumes at M0 were not predictive of changes in clinical outcomes, nor for changes in digital outcomes over 1 year (Fig. 4). Ventricle volume at M0 showed a trend for predicting FMSC change (std. *β* = –0.30, uncorrected *p* = 0.029), but did not survive correction for multiple comparisons. In addition, a trend was observed for thalamic volume predicting EDSS change (std. *β* = –0.25, uncorrected *p* = 0.048) and lesion volume for predicting s2MWT (std. *β* = –0.29, uncorrected *p* = 0.055).

## Discussion

Our findings reveal cross-sectional associations between digital outcome measures (sSDMT, FMSC and CSC) and brain atrophy measures and lesion volumes. The strongest cross-sectional relations with MRI volumes were found for the passively collected smartphone keystroke dynamics (FMSC and CSC). From the clinical outcome measures, SDMT demonstrated the strongest cross-sectional relations to brain atrophy and lesion volumes, while NHPT was related to lesion volumes only. No longitudinal associations between brain atrophy and digital- nor clinical outcome measures were found over a 1-year follow-up.

Our results indicate cross-sectional relations between the digital (CSC, sSDMT) and clinical (SDMT) outcome measures that aim to monitor cognition in PwMS, and thalamic and lesion volume. These findings are in line with numerous preceding MRI studies that show an association between SDMT performance and MRI markers of tissue damage, with the thalamus as the most relevant GM structure [34, 35]. As earlier investigations in this cohort demonstrated a correlation between the SDMT and sSDMT, the similar associations to thalamic atrophy and lesion volume were expected, although it should be noted that the conventional SDMT exhibited slightly stronger relations to the MRI measures compared to the sSDMT [16]. The relation between sSDMT and brain volume was weak, while a strong association was found between the conventional SDMT and brain volume. This is in contrast to a previous study showing high relations between a smartphone-based SDMT and brain atrophy [23]. Additionally, the CSC was similarly related to global MRI measures of atrophy (brain and ventricle volume) compared to the SDMT, which could also be expected given the association between SDMT and CSC demonstrated in an earlier study [17]. It should be noted that we did not convert SDMT scores to Z-scores, due to the absence of normative data for sSDMT and CSC, which should be explored in future research.

The FMSC demonstrated strong associations with brain, ventricle, thalamic and lesion volumes, aligning with our CSC findings. These results suggest a potential link between arm function and MRI volumes, as the FMSC, a measure for manual dexterity, has previously shown associations with worse arm function (NHPT) [17]. However, our study did not reveal significant NHPT associations with MRI volumes, except for lesion volumes. Although we found a trend for thalamic atrophy in relation to NHPT score, previous studies showed stronger relationships between NHPT performance and widespread structural GM damage in mainly motor- and cognitive related areas [36, 37]. This discrepancy can be explained by the fact that our sample was relatively small and more homogeneous compared to the other studies. The closer relation between FMSC and MRI volumes compared to NHPT might indicate that the FMSC is able to pick up more subtle changes in upper limb function, by measuring arm function in day-to-day life passively (FMSC) instead of concentrating on one manual task (NHPT).

No cross-sectional relations with brain volumes were found for the EDSS and T25FW, nor the s2MWT. In addition, no difference in s2MWT was found between PwMS and controls, which could indicate the lack of sensitivity for subtle walking dysfunction of this measure. The results for the EDSS and T25FW are somewhat unexpected, since cross-sectional correlations between these clinical disability outcome measures and MRI volume measures have been extensively described in the previous literature [38–43]. As it is expected that higher rates of disability are increasingly related to volume measures, this discrepancy with our results might be due to the relatively low impairment in our sample (median EDSS 3.5, IQR: 2.5–4.0) [42, 44]. In addition, spinal cord lesions and -atrophy were not taken into account in this study, which is an important driver of walking dysfunction in long-standing disease [45].

No longitudinal relations were found between brain or lesion volume and digital outcomes, nor with clinical outcomes, probably due to the relatively short follow-up of 1 year and subtle clinical changes. Although we observed significant changes in MRI volumes for the ventricles, thalamus and total brain volumes, a 1-year follow-up may not be sufficient to robustly measure neurodegeneration due to the measurement noise in combination with the relatively slow neurodegenerative process [46]. Moreover, the SDMT and digital outcome measures showed an improvement over time, indicating practice effects in these measures. Practice effects of clinical and digital outcomes, and especially cognitive testing (both SDMT and sSDMT), are increasingly recognized and are an area of concern when trying to capture progression, indicating the need for new ways to correct longitudinal changes [19, 47] [48]. Interestingly, prior research shows that the practice effect on the sSDMT is most distinct in the first three months of measurements and that after this a plateau phase is reached, although it differs between patients [19]. The present study indicates a practice effect on all digital outcomes. As a result, the baseline values used in this study might be an underestimation of the true performance of patients. A future direction should be to establish a new post-practice baseline value, to bypass the learning effect that digital outcomes measures face.

### Limitations

The main limitation of our study was the relatively short follow-up duration of 1 year, which may not be sufficient to assess disease worsening and neurodegeneration [49]. Although we found robust cross-sectional relations between digital outcomes and brain volume, investigating longitudinal relations over a longer follow-up would be of high interest. In addition, the sample size of our HC group was relatively small and no HC digital nor MRI data were available at follow-up. Hence, we were unable to correct for practice effects longitudinally and to disentangle disease-related neurodegenerative changes from normal ageing. Finally, another issue with digital and high-frequent monitoring is the adherence of using these applications. For example, the relatively high rates of missing data for the s2MWT may be attributed to the necessity to conduct this test outside, creating an extra burden for the patients. For the passive collection of CSC and FMSC, patients need to get used to an alternative smartphone keyboard which might act differently than their default one. Moreover, our missing data analyses showed that subjects with lack of adherence to passive keystroke dynamics collection were older and showed worse SDMT scores compared to the rest of the sample. This indicates that adherence might differ between certain types of patients. To be able to gain further insight in adherence, future research is needed to examine the user experience of the different digital health technologies.

## Conclusion

In conclusion, our findings further strengthen the evidence that digital biomarkers are relevant in MS due to their association with MRI-derived brain volumes. Strongest associations with brain volumes were found for measures of smartphone-based keystroke dynamics and the smartphone-based SDMT. These associations were similarly found by the conventional SDMT, but not consistently present for the other clinical outcomes (EDSS, T25FW and NHPT). Therefore, digital monitoring tools could provide new information on disease progression, indicating the need for future validation in additional longitudinal cohorts.

## Supplementary Information

Below is the link to the electronic supplementary material.

Supplementary file1 (DOCX 40 KB)
