# Supplementary material for: Digital outcome measures are associated with brain atrophy in patients with multiple sclerosis
Source: J Neurol. 2024 Jul 15;271(9):5958–68. doi: 10.1007/s00415-024-12516-9 (PMC11377687; doi:10.1007/s00415-024-12516-9)
Supplement: Supplementary file 1 — Supplementary file1 (DOCX 40 KB) [file 415_2024_12516_MOESM1_ESM.docx]

**Supplementary materials**

**Missing data analyses**

*Methods*

Missing data analyses were performed for each digital monitoring tool separately at M0 and M12, to see if patients with missing data differed from PwMS with complete data. Demographical- (age, gender, MS subtype, duration of disease) and clinical (EDSS, T25FW, 9HPT, SDMT) outcomes were compared using an independent samples t-test for normally distributed continuous variables, a Mann Whitney U test for non-normally distributed continuous variables, a Fisher exact test for binary variables and a Fisher-Freeman-Halton test for categorical variables with more than two levels. Statistical testing was only performed if more than 5% of patients had missing data.

*Results*A missing data analyses was performed for the sSDMT and s2MWT at both M0 and M12. As there was <5% missing data for PwMS on the CSC and FMSC at M0, we only performed a missing data analysis on M12 for these outcomes. PwMS with missing M0 sSDMT data had a higher EDSS (3.5 vs. 3.0) at M0, while PwMS with missing M12 sSDMT data had lower T25FW scores (4.87 vs. 5.79 seconds). PwMS of which s2MWT data at M0 was missing, were older (50.2 vs. 45.9 years) and had higher EDSS scores (4.0 vs. 3.0). PwMS with missing CSC and FMSC scores at M12 were older (52.2 vs. 45.6) and had lower M12 sSDMT scores (56.1 vs. 63.1). All other demographical and clinical variables did not differ significantly between PwMS with missing digital outcome data and the ones with complete digital outcome data. The full results from the missing data analysis can be found in supplementary tables 1, 2, 3 and 4*.*

| **sSDMT** | | | | | | |
| --- | --- | --- | --- | --- | --- | --- |
|  | **Available M0** | **Missing M0** | **p-value** | **Available M12** | **Missing M12** | **p-value** |
|  | (N=85) | (N=7) |  | (N=63) | (N=12) |  |
| **Age (years)** |  |  |  |  |  |  |
| Mean (SD) | 47.1 (10.1) | 43.3 (14.2) | 0.51 | 47.5 (10.1) | 46.0 (11.5) | 0.69 |
| **Gender** |  |  |  |  |  |  |
| Male | 23 (27.1%) | 2 (28.6%) | 1 | 20 (31.7%) | 3 (25.0%) | 0.75 |
| Female | 62 (72.9%) | 5 (71.4%) |  | 43 (68.3%) | 9 (75.0%) |  |
| **MS subtype** |  |  |  |  |  |  |
| RRMS | 50 (58.8%) | 5 (71.4%) | 0.94 | 33 (52.4%) | 9 (75.0%) | 0.24 |
| SPMS | 25 (29.4%) | 2 (28.6%) |  | 20 (31.7%) | 3 (25.0%) |  |
| PPMS | 10 (11.8%) | 0 (0%) |  | 10 (15.9%) | 0 (0%) |  |
| **Duration of disease (years)** |  |  |  |  |  |  |
| Median [IQR] | 6.47 [11.1] | 4.16 [2.36] | 0.21 | 6.18 [11.9] | 6.49 [4.67] | 0.98 |
| **EDSS** |  |  |  |  |  |  |
| Median [IQR] | 3.50 [1.50] | 3.00 [1.00] | 0.04 | 3.50 [2.00] | 2.75 [2.00] | 0.16 |
| **T25FW (seconds)** |  |  |  |  |  |  |
| Mean (SD) | 5.40 (2.08) | 5.19 (1.58) | 0.77 | 5.79 (2.61) | 4.87 (0.903) | 0.04 |
| **9-HPT (seconds)** |  |  |  |  |  |  |
| Mean (SD) | 22.7 (6.24) | 22.7 (4.27) | 0.99 | 22.1 (6.90) | 21.0 (3.90) | 0.45 |
| **SDMT** |  |  |  |  |  |  |
| Mean (SD) | 55.0 (10.2) | 52.6 (8.00) | 0.47 | 60.9 (11.8) | 63.9 (16.7) | 0.57 |

Supplementary table 1. Missing data analyses comparing demographical and clinical outcome values between PwMS with sSDMT available and PwMS with sSDMT missing for baseline (M0) and follow-up (M12).

*Abbreviations: sSDMT = smartphone-based SDMT, PwMS = People with Multiple Sclerosis, RRMS = relapsing-remitting multiple sclerosis, SPMS = secondary progressive multiple sclerosis, PPMS = primary progressive multiple sclerosis, EDSS=expanded disability status scale, T25FW=Timed 25 Foot Walk test, 9HPT=9-hole peg test, SDMT=Symbol Digit Modalities Test.*

| **s2MWT** | | | | | | |
| --- | --- | --- | --- | --- | --- | --- |
|  | **Available M0** | **Missing M0** | **p-value** | **Available M12** | **Missing M12** | **p-value** |
|  | (N=74) | (N=18) |  | (N=48) | (N=27) |  |
| **Age (years)** |  |  |  |  |  |  |
| Mean (SD) | 45.9 (11.0) | 50.2 (6.26) | 0.03 | 47.0 (10.6) | 47.6 (9.86) | 0.80 |
| **Gender** |  |  |  |  |  |  |
| Male | 23 (31.1%) | 2 (11.1%) | 0.14 | 15 (31.3%) | 8 (29.6%) | 1 |
| Female | 51 (68.9%) | 16 (88.9%) |  | 33 (68.8%) | 19 (70.4%) |  |
| **MS subtype** |  |  |  |  |  |  |
| RRMS | 45 (60.8%) | 10 (55.6%) | 1 | 26 (54.2%) | 16 (59.3%) | 0.94 |
| SPMS | 21 (28.4%) | 6 (33.3%) |  | 15 (31.3%) | 8 (29.6%) |  |
| PPMS | 8 (10.8%) | 2 (11.1%) |  | 7 (14.6%) | 3 (11.1%) |  |
| **Duration of disease (years)** |  |  |  |  |  |  |
| Median [IQR] | 6.42 [11.0] | 5.53 [6.37] | 0.89 | 6.05 [13.9] | 6.18 [6.55] | 0.97 |
| **EDSS** |  |  |  |  |  |  |
| Median [IQR] | 3.0 [1.50] | 4.0 [2.25] | 0.04 | 3.50 [1.50] | 4.00 [3.75] | 0.37 |
| **T25FW (seconds)** |  |  |  |  |  |  |
| Mean (SD) | 5.25 (1.84) | 5.97 (2.72) | 0.31 | 5.38 (2.27) | 6.12 (2.71) | 0.25 |
| **9-HPT (seconds)** |  |  |  |  |  |  |
| Mean (SD) | 22.5 (5.98) | 23.4 (6.67) | 0.61 | 21.3 (3.48) | 22.9 (9.84) | 0.43 |
| **SDMT** |  |  |  |  |  |  |
| Mean (SD) | 54.8 (10.5) | 55.1 (7.94) | 0.90 | 61.6 (13.8) | 61.1 (10.3) | 0.88 |

Supplementary table 2. Missing data analyses comparing demographical and clinical outcome values between PwMS with s2MWT available and PwMS with sSDMT missing for baseline (M0) and follow-up (M12).

*Abbreviations: s2MWT = smartphone-based 2 Minute Walk Test, PwMS = People with Multiple Sclerosis, RRMS = relapsing-remitting multiple sclerosis, SPMS = secondary progressive multiple sclerosis, PPMS = primary progressive multiple sclerosis, EDSS=expanded disability status scale, T25FW=Timed 25 Foot Walk test, 9HPT=9-hole peg test, SDMT=Symbol Digit Modalities Test.*

| **CSC** | | | | | | |
| --- | --- | --- | --- | --- | --- | --- |
|  | **Available M0** | **Missing M0** | **p-value** | **Available M12** | **Missing M12** | **p-value** |
|  | (N=90) | (N=2) |  | (N=57) | (N=18) |  |
| **Age (years)** |  |  | - |  |  |  |
| Mean (SD) | 46.8 (10.3) | 48.0 (21.2) |  | 45.6 (10.5) | 52.2 (7.90) | 0.01 |
| **Gender** |  |  | - |  |  |  |
| Male | 25 (27.8%) | 0 (0%) |  | 18 (31.6%) | 5 (27.8%) | 1 |
| Female | 65 (72.2%) | 2 (100%) |  | 39 (68.4%) | 13 (72.2%) |  |
| **MS subtype** |  |  | - |  |  |  |
| RRMS | 54 (60.0%) | 1 (50.0%) |  | 33 (57.9%) | 9 (50.0%) | 0.79 |
| SPMS | 27 (30.0%) | 0 (0%) |  | 17 (29.8%) | 6 (33.3%) |  |
| PPMS | 9 (10.0%) | 1 (50.0%) |  | 7 (12.3%) | 3 (16.7%) |  |
| **Duration of disease** (years) |  |  | - |  |  |  |
| Median [IQR] | 5.96 [10.4] | 6.13 [4.08] |  | 5.34 [9.67] | 8.51 [11.1] | 0.13 |
| **EDSS** |  |  | - |  |  |  |
| Median [IQR] | 3.50 [1.50] | 3.75 [2.25] |  | 3.00 [1.50] | 4.00 [1.50] | 0.17 |
| **T25FW (seconds)** |  |  | - |  |  |  |
| Mean (SD) | 5.36 (2.00) | 6.43 (4.77) |  | 5.58 (2.64) | 5.82 (1.70) | 0.67 |
| **9-HPT (seconds)** |  |  | - |  |  |  |
| Mean (SD) | 22.7 (6.14) | 21.7 (4.76) |  | 22.1 (7.25) | 21.2 (3.22) | 0.45 |
| **SDMT** |  |  | - |  |  |  |
| Mean (SD) | 55.0 (10.1) | 46.0 (1.41) |  | 63.1 (13.0) | 56.1 (9.62) | 0.02 |

Supplementary table 3. Missing data analyses comparing demographical and clinical outcome values between PwMS with CSC available and PwMS with sSDMT missing for baseline (M0) and follow-up (M12). Given the low amount of missing CSC values at baseline no statistical tests were performed for the baseline values.

*Abbreviations: CSC = Cognition Score Cluster, PwMS = People with Multiple Sclerosis, RRMS = relapsing-remitting multiple sclerosis, SPMS = secondary progressive multiple sclerosis, PPMS = primary progressive multiple sclerosis, EDSS=expanded disability status scale, T25FW=Timed 25 Foot Walk test, 9HPT=9-hole peg test, SDMT=Symbol Digit Modalities Test.*

| **FMSC** | | | | | | |
| --- | --- | --- | --- | --- | --- | --- |
|  | **Available M0** | **Missing M0** | **p-value** | **Available M12** | **Missing M12** | **p-value** |
|  | (N=91) | (N=1) |  | (N=57) | (N=18) |  |
| **Age (years)** |  |  | - |  |  |  |
| Mean (SD) | 46.9 (10.3) | 33.0 |  | 45.6 (10.5) | 52.2 (7.90) | 0.01 |
| **Gender** |  |  | - |  |  |  |
| Male | 25 (27.5%) | 0 (0%) |  | 18 (31.6%) | 5 (27.8%) | 1 |
| Female | 66 (72.5%) | 1 (100%) |  | 39 (68.4%) | 13 (72.2%) |  |
| **MS subtype** |  |  | - |  |  |  |
| RRMS | 54 (59.3%) | 1 (100%) |  | 33 (57.9%) | 9 (50.0%) | 0.79 |
| SPMS | 27 (29.7%) | 0 (0%) |  | 17 (29.8%) | 6 (33.3%) |  |
| PPMS | 10 (11.0%) | 0 (0%) |  | 7 (12.3%) | 3 (16.7%) |  |
| **Duration of disease** (years) |  |  | - |  |  |  |
| Median [IQR] | 6.18 [10.3] | 2.05 [0] |  | 5.34 [9.67] | 8.51 [11.1] | 0.13 |
| **EDSS** |  |  |  |  |  |  |
| Median [IQR] | 3.50 [1.50] | 1.50 [0] |  | 3.00 [1.50] | 4.00 [1.50] | 0.17 |
| **T25FW (seconds)** |  |  | - |  |  |  |
| Mean (SD) | 5.41 (2.04) | 3.05 |  | 5.58 (2.64) | 5.82 (1.70) | 0.66 |
| **9-HPT (seconds)** |  |  | - |  |  |  |
| Mean (SD) | 22.7 (6.11) | 18.33 |  | 22.1 (7.25) | 21.2 (3.22) | 0.47 |
| **SDMT** |  |  | - |  |  |  |
| Mean (SD) | 54.9 (10.1) | 47.0 |  | 63.1 (13.0) | 56.1 (9.62) | 0.02 |

Supplementary table 4. Missing data analyses comparing demographical and clinical outcome values between PwMS with FMSC available and PwMS with sSDMT missing for baseline (M0) and follow-up (M12). Given the low amount of missing CSC values at baseline no statistical tests were performed for the baseline values.

*Abbreviations: FMSC = Fine Motor Score Cluster, PwMS = People with Multiple Sclerosis, RRMS = relapsing-remitting multiple sclerosis, SPMS = secondary progressive multiple sclerosis, PPMS = primary progressive multiple sclerosis, EDSS=expanded disability status scale, T25FW=Timed 25 Foot Walk test, 9HPT=9-hole peg test, SDMT=Symbol Digit Modalities Test.*
